# Why is it necessary to validate models of pedagogical competency?

**DOI:** 10.3205/zma001124

**Published:** 2017-10-16

**Authors:** Niclas Schaper

**Affiliations:** 1 Universität Paderborn, Fakultät für Kulturwissenschaften, Institut für Humanwissenschaften, Lehrstuhl für Arbeits- und Organisationspsychologie, Paderborn, Germany

## Short report

In recent years many different forms of competency models have been used in curricular design, quality assurance and education research. This trend has also been addressed and reinforced in higher education, particularly within the framework of the Bologna Reform (in terms of competency-based learning). Competency models now play a role in the education and professional advancement of university teachers. These models indicate which skills teachers need in different subjects in order to effectively prepare students for professional careers in science and other academic fields. Many different approaches and models have been generated offering systematic, competency-based descriptions of the professional teaching skills necessary for university-level educators. Suitable pedagogical competency models are needed for different purposes and functions. Some examples include models to (vgl. [1])

- systematically train teachers in a targeted manner;
- systematically plan and impart theory-based approaches, such as problem-based learning;
- implement quality assurance and management of teaching in a targeted manner;
- make the educational objectives and requirements transparent for aspiring university teachers and others participating in the educational process.

There are now a series of approaches for describing teaching skills that are helpful in defining content elements and qualification dimensions, imparting the theoretical basis underlying the study program, and ensuring a systematic strategic approach and appropriate quality management (Trautwein & Merkt, 2013; [1]). However, the scientific caliber of such models varies. The models refer to different interpretations of the competency construct (e.g. the distinction between professional expertise, methodological competence, social skills and interpersonal skills or the definition of competency according to Weinert, 2001; [7]) or the models have different degrees of differentiation and identification of basic competencies, or even different modes of model construction (e.g. deductive vs. inductive; [6]). The central weakness seen in all common models is predominantly the lack of model validation regarding their theoretical and conceptual validity and regarding their specific purposes.

The first issue to arise in this context is why should competency models be tested regarding their validity, especially if the basis for their inferences is, in many respects, normatively justified? It must be pointed out here that competency models as (psychological) constructs must have a conclusive theoretical basis and, like other (psychological) constructs, must be supported by evidence. To ensure this, suitable strategies can be used for validation, which are not only relevant for the psychometric examination of test instruments [2], [8]. In my opinion, the following criteria must be applied to validate such models. *First*, the models should be based on proven and evidence-based notions about the structure and ranking of skills in a field of application (including the underlying concept of competence). *Second,* they should be consistent and generalizable in their descriptions of competencies for a particular professional domain. *Third*, their conclusions concerning practical applicability should be based on theoretically and empirically supported evidence and arguments. *Fourth*, competency models should also be organized and formulated such that they can be understood by the target groups while making reference to needs and prior conceptions to ensure sufficient acceptance within the target group. Only if these criteria are adequately met it can be assumed that such competency models actually generate new insights, that they can be justifiably used for the intended purpose, and that they truly are suitable for improving teaching quality, whatever the educational context may be.

Validity in this context requires a broader understanding of what is needed for validity than what is called for by classic test theory. It does not only involve the determination of statistical values regarding construct and criteria validity, but also a modern understanding of validity in terms of the argument-based validation approach put forth by Messick [5] and Kane [4]. Both of these authors propose six different criteria or aspects of validation:

1. content validity, meaning the curricular and/or theoretical basis for the modeled area;
2. cognitive validity, meaning the matching of cognitive processes when measuring competencies to the postulated theoretical model of competency;
3. structural validity, meaning the matching of the theoretical competency model with the selected psychometric measurement model,
4. generalizability, meaning the appropriateness of an interpretation of the model going beyond the specific groups of tasks and people;
5. external validity, meaning commensurateness with other constructs in regard to convergent, discriminant and predictive correlations;
6. consequential validity, meaning the appropriateness of the interpretation of results and the resulting consequences.

In regard to generating and applying (pedagogical) competency models for qualification purposes, content and consequential validity, in particular, are important. Playing a secondary role are cognitive validity, external validity, and the validation criterion for generalizability. Justifications as to why these specific aspects should be considered when validating competency models and what should be heeded are addressed in the following:

The *content validity* of competency models is of special significance since this form of validation clarifies whether a model does the best possible job representing the characteristics or behavior of interest within a particular competency domain from a theoretical and content-oriented practical perspective. To verify the content validity of (pedagogical) competency models, the current academic research on the construct is analyzed in detail and applied to the model in terms of its definitions of terms and structure (e.g. does the conceptual model refers to existing and proven model designs?). When validating content, experts (e.g. those who educate and train university teachers) are also asked to evaluate the relevance and aptness of the aspects and dimensions of competency described by the model. In addition, experts are also able to rate if the subject-specific particularities/requirements connected with a competency domain have been sufficiently accounted for.

The* cognitive validity* of a competency model focuses on the thoughts and actions described in the model which are necessary within the domain for competent professional decision making. As an expansion of the content validity this validity aspect entails not only the structure and content of the relevant skills, but also if the model also adequately describes relevant processes, for instance, the application of knowledge. To assess this, classroom observations and teacher surveys can be carried out regarding university teachers’ planning activities and reflections on their actions. When doing this, it is critical to verify if the knowledge and skills described by the model are relevant to planning, implementing, assessing and reflecting on teaching activities.

Testing for *external validity* is associated with another central issue pertaining to the effectiveness of the competency model. The model is examined to verify if it sufficiently identifies and describes the skills relevant to success in designing effective teaching. Generally the teaching skills described in the model are measured using tests, self-assessment, or assessment by another and then they are correlated with outcome variables such as knowledge gain of the instructed students or ratings of teaching quality [8]. Studies and analyses of external validity permit conclusions to be drawn or evidence-based statements to be made regarding the efficacy or predictability of the effects of the particular (teaching) skills.

When verifying the *consequential validity* of competency models, it is important to examine the match between the competency model on the one side and statements concerning the intended use that can be made on its basis on the other side. When dealing with pedagogical competency models it is important to clarify if the conclusions and inferences made in regard to learning objectives and implementation strategies in university teacher training and education have been sufficiently based on theory (e.g. refer to training approaches based on theories of learning) and if the conclusions can be inferred or accounted for on the basis of empirical evidence (if, for instance, the efficacy of the training could be shown using evaluative studies). This can be done, for example, by carrying out a detailed conceptual analysis or surveying education experts in the (subject-specific) teaching program and teachers of the subject itself. The latter is meant to assess the extent to which the practical conclusions appear apt to the experts and to address and account for practical needs.

The ability to *generalize competency* models refers to the question, to what extent the skills descriptions can be applied beyond the various contexts and target groups identified in a given model? To evaluate this validity criterion in pedagogical competency models it is important to clarify the extent to which a competency model with all of its different facets can be transferred to other or related contexts in higher education (e.g. if the pedagogical competency model for medical education can be applied or transferred to different contexts in medical teaching). This could be tested, for instance, by applying the model to different target groups, teaching formats or subject combinations. Then it has to be evaluated concerning the extent to which the model categories sufficiently and appropriately reflect the different skills requirements and if the model’s structure does not significantly change when applied to different contexts.

Finally, I would like to briefly outline how these validation concepts can be applied to the competency model for teaching medicine described in the position paper by the GMA committee on human resource and organizational development in academic teaching (KLM; [3]):

1. In reference to the *content validity* of the KLM model, it has already been verified to what extent the model conforms and diverges from other subject-specific competency models for teaching medicine (vgl. [3]). In a further validation step, I would recommend theoretical scrutiny of the model to ascertain the extent to which the KLM corresponds with general pedagogical competency models drawn from the field of academic teaching, which dimensions are corroborated, which are not taken into account, and if additional structural elements (e.g. levels of competency) are considered. From this it is possible to draw conclusions about the content-based and conceptual development of the model.
2. In reference to *cognitive validity*, I would propose that classroom observations in different, yet typical, medical education settings are conducted to see to what extent the KLM describes the main aspects of competent teaching and if the perceptions and evaluations of teaching situations are described in a sufficiently differentiated manner by teachers of medicine at the university level. This enables improved and precise definition of the practical nature of the skill descriptions.
3. In reference to an *external validation* of the KLM, I would suggest that the model should be examined in regard to its effectiveness in predicting good teaching outcomes and teaching success. Initially, an instrument to measure the central skills fields of the KLM needs to be developed (e.g. in the form of a scale for self-assessment or assessment by another person). Following that, it would be possible to apply evaluations of the different skills fields and their facets to predict teacher evaluations (by students or trained observers) and of teaching success (e.g. in the acquisition of competency by the students). This can serve to analyze and compare the predictive contribution, for instance, of the individual skills fields for the criteria of teaching quality and teacher success in order to get an indication of how to weight the measured competencies.
4. To verify the *consequential validity* of the KLM, it is recommended that the individual needs and measures regarding development should be evaluated in terms of relevance and adequacy using an expert survey (people with medical and pedagogical expertise). On the basis of this, it would be possible to improve the practical application of the model and the inferred statements and instruments.
5. Regarding the *generalizability of the model*, data on and comparisons of different teaching tasks and requirements and positions (e.g. differentiated according to study phase or subject combination in medical education) should be gathered. Based on this, it would be possible to analyze the extent to which the KLM model aptly captures and reflects the teacher requirements and success-related skills and competencies.

In conclusion, it must be emphasized that properly validating competency models is not only important in medical teaching, but also in other university teaching contexts found in higher education where there is also a need for this. Finally, the validation approaches described here pose an opportunity to implement and advance the knowledge and insights gained regarding theory and practice and in relation to the design of programs in academic teaching and their quality assurance.

## Competing interests

The author declares that he has no competing interests. 
